# Reduction of porcine circovirus type 2 (PCV2) viremia by a reformulated inactivated chimeric PCV1-2 vaccine-induced humoral and cellular immunity after experimental PCV2 challenge

**DOI:** 10.1186/1746-6148-8-194

**Published:** 2012-10-19

**Authors:** Hwi Won Seo, Yeonsu Oh, Kiwon Han, Changhoon Park, Chanhee Chae

**Affiliations:** 1 College of Veterinary Medicine, Department of Veterinary Pathology, Seoul National University, 599 Gwanak-ro, Gwanak-gu, Seoul, 151–742, Republic of Korea

**Keywords:** Chimeric PCV1-2 vaccine, Efficacy, Humoral and cellular immunity, Porcine circovirus-associated disease, Porcine circovirus type 2, Porcine circovirus vaccine

## Abstract

**Background:**

The objective of the present study was to elucidate the humoral and cellular immune response mechanisms by which a reformulated inactivated chimeric PCV1-2 vaccine reduces the PCV2 viremia. Forty PCV2 seronegative 3-week-old pigs were randomly divided into the following four groups: vaccinated challenged (T01), vaccinated non-challenged (T02), non-vaccinated challenged (T03), and non-vaccinated non-challenged (T04) animals. The pigs in groups T01 and T02 were immunized with a reformulated inactivated chimeric PCV1-2 vaccine (Fostera™ PCV; Pfizer Animal Health) administered as a 2.0 ml dose at 21 days of age. At 35 days of age (0 days post-challenge), the pigs in groups T01 and T03 were inoculated intranasally with 2 ml each of PCV2b.

**Results:**

A reduction of PCV2 viremia coincided with the appearance of both PCV2-specific neutralizing antibodies (NA) and interferon-γ-secreting cells (IFN-γ-SCs) in the vaccinated animals. However, the presence of anti-PCV2 IgG antibodies did not correlate with the reduction of PCV2 viremia. Lymphocyte subset analysis indicated that the numbers of CD3^+^ and CD4^+^ cells increased in vaccinated animals but the numbers of CD4^+^ cells decreased transiently in non-vaccinated animals. The observation of a delayed type hypersensitivity response in only the vaccinated animals also supports a CD4^+^ cell-associated protective cellular immune response induced by the reformulated inactivated chimeric PCV1-2 vaccine.

**Conclusions:**

The induction of PCV2-specific NA and IFN-γ-SCs, and CD4^+^ cells by the reformulated inactivated chimeric PCV1-2 vaccine is the important protective immune response leading to reduction of the PCV2 viremia and control of the PCV2 infection. To our knowledge this is the first demonstration of protective humoral and cellular immunity induced by the reformulated inactivated chimeric PCV1-2 vaccine and its effect on reduction of PCV2 viremia by vaccination.

## Background

Porcine circovirus type 2 (PCV2) is one of the most economically important swine pathogens worldwide. The first commercial PCV2 vaccine was used under special license in France and Germany in 2004, 13 years after postweaning multisystemic wasting syndrome (PMWS) was first identified and reported in western Canada [1]. In addition to PMWS, PCV2 is also associated with a number of diseases and syndromes, collectively referred to as porcine circovirus-associated disease (PCVAD) [2,3].

Currently, 5 commercial PCV2 vaccines are available worldwide and differ in their antigen [1]. One vaccine (Circovac, Merial) is based on the classical approach of an inactivated oil-adjuvanted vaccine. Three subunit vaccines (Circoflex, Boehringer Ingelheim; Circumvent PCV, Intervet/Merck; Porcillis PCV, Schering-Plough/Merck) are based on an open reading frame 2 (ORF2; capsid) protein expressed in the baculovirus system. Another vaccine (Suvaxyn PCV2 One Dose, Pfizer Animal Health/Fort Dodge Animal Health) is based on a chimeric PCV1-2 virus containing the genomic backbone of the non-pathogenic PCV1, with the ORF2 capsid gene replaced by that of PCV2 [4]. In 2008, Pfizer Animal Health temporarily removed the inactivated chimeric PCV1-2 vaccine product from the markets because a chimeric PCV1-2 virus was incidentally detected in the field due to incomplete inactivation of the vaccine [5]. In August 2011, a reformulated version of the chimeric PCV1-2 vaccine under a new brand name (Fostera™ PCV, Pfizer Animal Health) re-entered the market.

PCV2 viremia plays a central role in the development of PMWS. High levels of PCV2 viremia are associated with the development of PCVAD [6,7]. Reductions in the PCV2 viremia have coincided with the appearance of both PCV2-specific neutralizing antibodies (NA) and interferon γ-secreting cells (IFN-γ-SCs) in PCV2-infected animals [6-9]. Therefore, the induction of PCV2-specific NA and IFN-γ-SCs by commercial PCV2 vaccines is a critical parameter to evaluate the efficacy of PCV2 vaccines in the control of PCV2 infection. It has been reported that subunit and inactivated PCV2 vaccines elicit PCV2-specific NA and IFN-γ-SCs [10,11]. Although the former inactivated chimeric PCV1-2 vaccine induced PCV2-specific NA [12], little is known regarding the protective immunity by which the reformulated inactivated chimeric PCV1-2 vaccine reduces the PCV2 viremia. The objective of present study is to elucidate the mechanisms how humoral and cellular immune response induced by the reformulated inactivated chimeric PCV1-2 vaccine reduces PCV2 viremia under experimental conditions.

## Methods

### Animals and housing

A total of 40 colostrum-fed, cross-bred, conventional piglets were purchased at 14 days of age from a commercial farm. All piglets were negative for porcine reproductive and respiratory syndrome virus (PRRSV) and *Mycoplasma hyopneumoniae* according to routine serological testing. All piglets were negative for PCV1-2a and PCV2 viremia by real-time polymerase chain reaction (PCR), respectively [13,14]. All piglets were also seronegative against PCV2 according to commercial ELISA (Synbiotics, Lyon, France). All pigs were housed in an environmentally controlled building as previously described [15].

### Experimental design

A total of 40 piglets were randomly divided into 4 groups (10 pigs per group). The pigs in groups T01 and T02 were immunized with an inactivated chimeric PCV1-2 vaccine (Fostera™ PCV; Pfizer Animal Health Inc.) administered as a 2.0 ml dose at 21 days of age based on the manufacturer’s recommendations. At 35 days of age [0 days post-challenge (dpc)], the pigs in groups T01 and T03 were inoculated intranasally with 2 ml each of a PCV2b [strain SNUVR000463; 5th passage; 1.0 × 10^5^ tissue culture infective dose of 50% (TCID50)/ml]. The pigs in group T04 remained unvaccinated and unchallenged, and served as the negative control group. The pigs in each group were housed separately within the facility. Blood samples and nasal swabs were collected at −14, 0, 14, and 28 dpc. All pigs were euthanized for necropsy at 28 dpc and superficial inguinal lymph nodes were collected for histopathology and immunohistochemistry. All of the methods were previously approved by the Seoul National University Institutional Animal Care and Use Committee.

### Quantification of PCV2 DNA in blood and nasal swab

DNA was extracted from serum and nasal samples using the QIAamp DNA mini kit. DNA extracts were used to quantify PCV2 genomic DNA copy numbers by real-time PCR as previously described by Gagnon et al. (2008) [14]. DNA extracts from serum samples were also used to detect PCV1-2a DNA by real-time PCR as previously described by Shen et al. (2010) [13].

### Serology

The serum samples were tested using the commercial PCV2 ELISA IgG (Synbiotics, Lyon, France) and serum virus neutralization (SVN) test [16].

### Preparation of PCV2 antigen

The same PCV2 strain used for challenge in the present study, was propagated in PCV-free PK15 cells to a titer of 10^4^ TCID50/ml and treated with two freeze-thaw cycles. The PCV2 antigen was prepared by concentrating the virus present in cell culture by ultracentrifugation at 100,000x *g* at 4°C for 3 h. The virus pellet was resuspended with PBS. The concentrated PCV2 was inactivated by exposing to an 8 W germicidal UV lamp at a distance of 15 cm for 1 h. Immunoperoxidase assay was performed to confirm the inactivation of virus as previously described by Rodriguez-Arrioja et al. (2000) [17].

### Delayed type hypersensitivity

The delayed type hypersensitivity (DTH) test was performed on 40 piglets from 4 groups at 5 weeks of age (2 weeks after vaccination; 0 dpc). Piglets were injected intradermally on the left inguinal area with 250 μg of PCV2 antigen from infected PK15 cells. Phytohemaglutin (PHA; Roche Diagnostics GmbH; 20 μg/ml in 0.1 ml) and saline (0.1 ml) were used as positive and negative controls, respectively. The mean diameters of the induration at the test site were measured with a micrometer 36 h after injection.

### Direct immunofluorescence assay

Cryosections (about 7 μm in thickness) were prepared from skin biopsy specimens in DTH test. Sections were transferred to microscope slides treated with poly-L-lysine. The slides were fixed with acetone for 20 min and then air-dried. The slides were then covered with 50 μl of mouse anti-pig CD4a conjugated with R-phycoerythrin (R-PE) (1:100 dilution; SouthernBiotech, Birmingham, AL, USA) and incubated for 60 min at 37°C in a moisture chamber. After three washing steps with PBS, the slides were mounted in buffered glycerin for observation by fluorescence microscopy.

### Enzyme-linked immunospot (ELISPOT) assay

The numbers of PCV2-specific interferon-γ-secreting cells (IFN-γ-SCs) were determined in peripheral blood mononuclear cells (PBMCs) at −14, 0, 14, and 28 dpc as previously described [18]. Briefly, 100 μl containing 2×10^6^ PBMCs in RPMI 1640 medium supplemented with 10% fetal bovine serum (HyClone Laboratories, Inc., SelectScience, Bath, UK) were seeded into plates pre-coated with anti-porcine IFN-γ monoclonal antibody (5 μg/ml, MABTECH, Mariemont, OH, USA) and incubated with 100 μl of PCV2 antigen (20 μg/ml), phytohemagglutinin (10 μg/ml, Roche Diagnostics GmbH, Mannheim, Germany) as a positive control, or PBS as a negative control for 40 h at 37°C in a 5% humidified CO_2_ atmosphere. Then, the wells were washed five times with PBS (200 μl per well). Thereafter, procedure was followed by manufacturer’s instructions using a commercial ELISPOT assay kit (MABTECH, Mariemont, OH, USA). Spots on the membranes were read by an automated ELISpot reader (AID ELISpot Reader, AID GmbH, Strassberg, Germany). The results were expressed as the number of responding cells per million PBMCs.

### Flow cytometry

PBMCs were incubated with R-PE- or FITC-conjugated mouse monoclonal antibodies (antiswine CD3 [R-PE] and CD4 [R-PE and FITC]; SouthernBiotech, Birmingham, AL, USA) for 30 min at 4°C in the dark and washed twice with PBS containing 0.1% sodium azide and 0.1% bovine serum albumin. Cells stained with conjugated antibodies were resuspended immediately in supplemented RPMI 1640 medium. Cells were analyzed using a FACSCalibur flow cytometer (Becton Dickinson) as previously described by Sosa et al. (2009) [19].

### Histopathology and immunohistochemistry

For the morphometric analysis of histopathological lesion score and PCV2 antigen score in superficial inguinal lymph nodes were collected at necropsy. In each sample, three sections were randomly selected and examined “blindly” as previously described by Kim et al. (2011) [15]. The scores of lesions in lymph nodes ranged from 0 (No lymphoid depletion or granulomatous replacement) to 5 (severe lymphoid depletion and granulomatous replacement), and the PCV2 antigen scores were obtained by counting the number of PCV2 positive cells per unit area (0.25 mm^2^).

### Statistical analysis

Continuous data (DTH response, PCV2 DNA, PCV2 serology, PCV2-specific IFN-γ-SCs, and lymphocyte subsets) were analyzed with a one-way analysis of variance (ANOVA). If a one-way ANOVA was significant (*P* < 0.05), pairwise testing using Tukey’s adjustment was performed. Discrete data (lymphoid lesion score and PCV2 antigen score, and proportion of viremic pigs) were analyzed by Chi-square and Fisher’s exact test.

The Pearson’s correlation coefficient was used to assess the relationship among viremia, serum virus neutralization titer, PCV2-specific IFN-γ-SCs, and the Spearman’s rank correlation coefficient was used to assess lymphoid lesion score and PCV2 antigen score. A value of *p* < 0.05 was considered significant.

## Results

### PCV2 DNA in sera and nasal swabs

**Figure 1:**
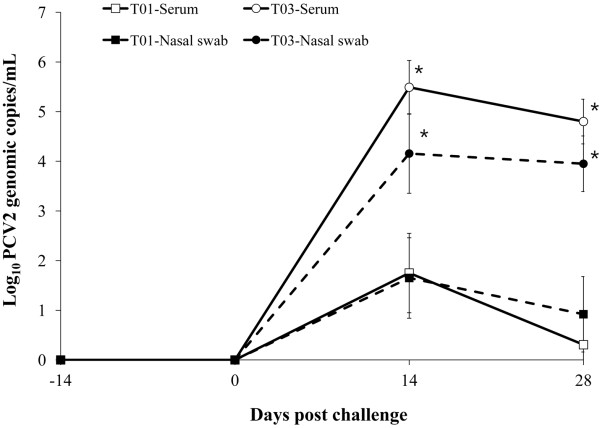
**Mean values of the genomic copy number of porcine circovirus type 2 DNA in serum and nasal swabs from vaccinated challenged (T01; □ for serum and ■ for nasal swab) and non-vaccinated challenged (T03; ◯ for serum and ● for nasal swab) animals.** Variation is expressed as the standard deviation. Significant difference is indicated at *p* value <0.001^*^.

PCV2 DNA was not detected in serum and nasal samples from any of pigs at 0 dpc. Vaccinated challenged (T01) animals had a significantly lower number of genomic copies of PCV2 in the blood than non-vaccinated challenged (T03) animals at 14 and 28 days post challenge (dpc; *p* < 0.001, Figure 1). The percentage of viremic pigs was significantly lower in vaccinated challenged animals (4/10 at 14 dpc and 2/10 at 28 dpc) compared to non-vaccinated challenged animals (10/10 at 14 and 28 dpc, *p* < 0.05). Vaccinated challenged animals had a significantly lower number of genomic copies of PCV2 in the nasal swab than non-vaccinated challenged animals at 14 and 28 dpc (*p* < 0.001, Figure 1). The percentage of nasal shedders was significantly lower in vaccinated challenged animals (4/10 at 14 and 28 dpc) than in non-vaccinated challenged animals (10/10 at 14 and 28 dpc, *p* < 0.05). The number of genomic copies of PCV2 in the blood correlated with that of PCV2 in the nasal swabs (T01: r^2^ = 0.921, *p* = 0.042 and T03: r^2^ = 0.972, *p* = 0.002). No PCV2 DNA was detected in the blood and nasal swabs from vaccinated non-challenged (T02) and non-vaccinated non-challenged (T04) animals throughout the experiment. No PCV1-2a DNA was detected in the blood from vaccinated (T01 and T02) and non-vaccinated (T03 and T04) animals by real-time PCR at −14, 0, 14, and 28 dpc.

### Anti-PCV2 IgG antibodies

At the time of PCV2 vaccination (3 weeks of age; -14 dpc), pigs in all 4 groups were seronegative against PCV2. Anti-PCV2 IgG antibody titers were significantly higher in vaccinated challenged (T01) and vaccinated non-challenged (T02) animals than in non-vaccinated challenged (T03) animals at 0, 14, and 28 dpc. Anti-PCV2 IgG antibody titers were not detected in non-vaccinated non-challenged (T04) animals throughout the experiment. Anti-PCV2 IgG antibody titers did not correlate with the number of genomic copies of PCV2 in the blood (T01: r^2^ = −0.332, *p* = 0.075 and T03: r^2^ = −0.105, *p* = 0.126).

### Neutralizing antibodies

**Figure 2:**
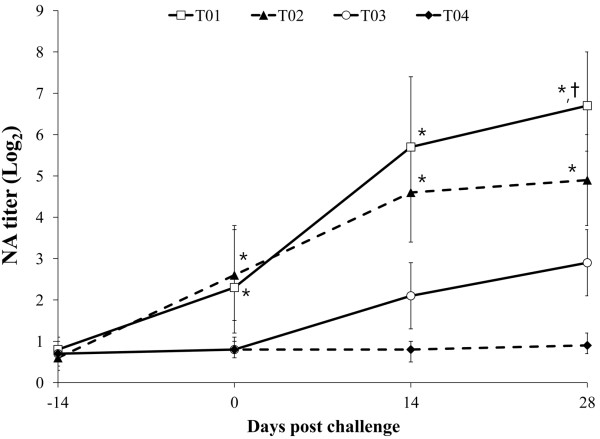
**Mean values of the serum neutralizing antibodies (NA) titer in the different groups; vaccinated challenged (T01; □), vaccinated non-challenged (T02; ▲), non-vaccinated challenged (T03; ○), and non-vaccinated non-challenged (T04; ♦) animals.** Variation is expressed as the standard deviation. Significant difference (T01 and T02 vs. T03 and T04) is indicated at *p* value <0.01^*^. Significant difference (T01 vs. T02) is indicated at *p* value <0.05^†^.

At the time of PCV2 vaccination (3 weeks of age, -14 dpc), no significant differences in NA titers were detected among the 4 groups. The NA titers were significantly higher in vaccinated challenged (T01) and vaccinated non-challenged (T02) animals than in non-vaccinated challenged (T03) animals group at 0, 14, and 28 dpc. The NA titers were not detected in non-vaccinated non-challenged (T04) animals throughout the experiment (Figure 2). The NA titers correlated inversely with the number of genomic copies of PCV2 in the blood (T01: r^2^ = −0.712, *p* = 0.012 and T03: r^2^ = −0.635, *p* = 0.031).

### PCV2-specific interferon- γ-secreting cells

**Figure 3:**
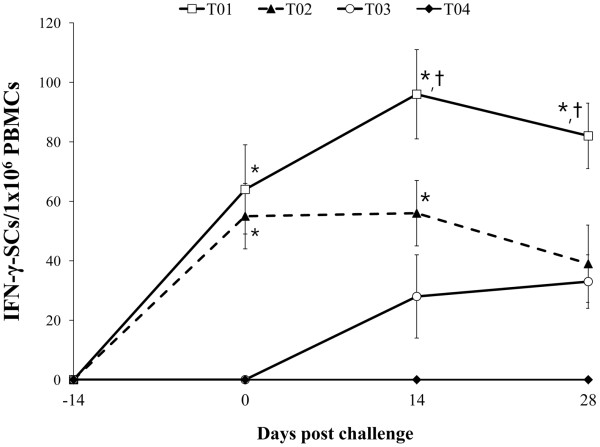
**Mean number of porcine circovirus type 2-specific interferon-γ-secreting cells (IFN-γ-SCs) in vaccinated challenged (T01; □), vaccinated non-challenged (T02; ▲), non-vaccinated challenged (T03; ○), and non-vaccinated non-challenged (T04; ♦) animals.** Variation is expressed as the standard deviation. Significant difference (T01 and T02 vs. T03 and T04) is indicated at *p* value <0.05^*^. Significant difference (T01 vs. T02) is indicated at *p* value <0.01^†^.

PCV2-specific IFN-γ-SCs were not observed in the isolated PBMCs of pigs from the 4 groups at −14 dpc. PCV2-specific IFN-γ-SCs increased sharply in vaccinated challenged (T01) and vaccinated non-challenged (T02) animals at 0 dpc (2 weeks after vaccination). At 0 and 14 dpc, the mean numbers of PCV2-specific IFN-γ-SCs were significantly higher in vaccinated challenged (T01) and vaccinated non-challenged (T02) animals compared to non-vaccinated challenged (T03) animals (*p* < 0.05). At 28 dpc, the mean numbers of PCV2-specific IFN-γ-SCs were significantly higher in vaccinated challenged (T01) than in vaccinated non-challenged (T02) and non-vaccinated challenged (T03) animals (*p* < 0.01). PCV2-specific IFN-γ-SCs were not detected in the PBMCs from non-vaccinated non-challenged (T04) animals throughout the experiment (Figure 3).

PCV2-specific IFN-γ-SCs also correlated inversely with the number of genomic copies of PCV2 in the blood (T01: r^2^ = −0.685, *p* = 0.022 and T03: r^2^ = −0.625, *p* = 0.028).

### Delayed type hypersensitivity

**Figure 4:**
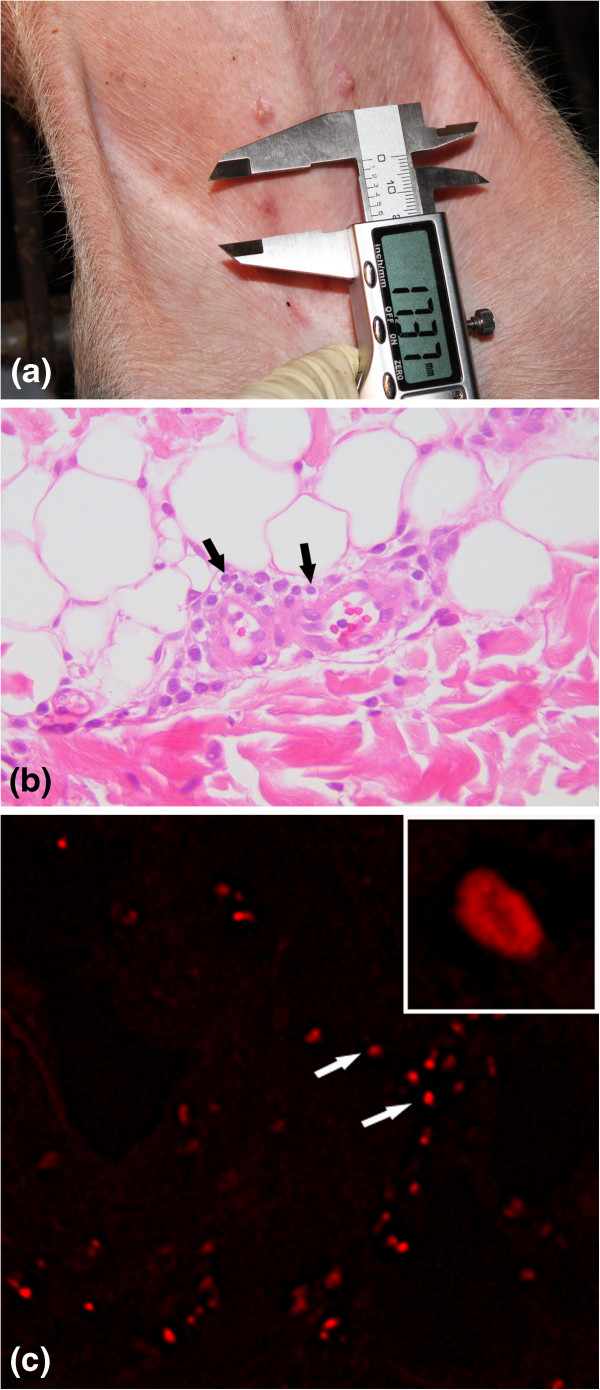
**Delayed type hypersensitivity induced by inactivated chimeric PCV1-2 vaccine in vaccinated challenged (T01) animals.** (**a**) erythematous lesion were grossly observed in the skin. (**b**) Perivascular infiltration of lymphocytes (black arrows) was seen in the dermis. (**c**) CD4^+^ cells (white arrows) were seen in the perivascular zone.

At 36 h after intradermal injection of the PCV2 antigen, vaccinated animals showed DTH responses consisted of induration and erythematous nodules (Figure 4a). Histopathologically, perivascular infiltrations of lymphocytes were observed in the dermis (Figure 4b). The specific fluorescence signals were observed in cells in perivascular zone (Figure 4c). The positive signals were mainly seen in the cytoplasm of cells.

The skin reaction regressed slowly at 48 h after injection and completely disappeared. DTH responses were not observed in the controls. The vaccinated animals in groups T01 and T02 and non-vaccinated animals in groups T03 and T04 showed DTH responses to the nonspecific mitogen PHA. The PHA DTH response size was not significantly different between vaccinated (T01 and T02) and non-vaccinated (T03 and T04) animals. The vaccinated (T01 and T02) animals displayed significantly greater PCV2-specific DTH responses than the non-vaccinated (T03 and T04) animals (*p* < 0.01, Table 1). DTH responses to saline injection were not observed in any pigs. DTH response correlated with PCV2-specific IFN-γ-SCs (T01: r^2^ = 0.637, *p* = 0.015 and T03: r^2^ = 0.219, *p* = 0.218).

**Table 1:** Mean number of delayed type hypersensitivity (DTH) response size, microscopic lymphoid lesion score and immunohistochemical Porcine circovirus type 2 (PCV2) antigen score in lymph node from vaccinated challenged (T01), vaccinated non-challenged (T02), non-vaccinated challenged (T03), and non-vaccinated non-challenged (T04) animals

	**Groups**			
	**T01**	**T02**	**T03**	**T04**
DTH	15.38^*^ ± 1.87	14.20^*^ ± 4.26	2.19 ± 0.08	1.98 ± 1.09
Lymphoid lesion score	0.40^†^ ± 0.54	-	1.67 ± 0.57	-
PCV2 antigen score	6.18^†^ ± 4.79	-	44.12 ± 9.07	-

^*^Significant difference (T01 and T02 vs. T03 and T04) is indicated at *p* value < 0.01. ^†^Significant difference (T01 vs. T03) is indicated at *p* value < 0.05.

### Identification of lymphocyte subsets

**Figure 5:**
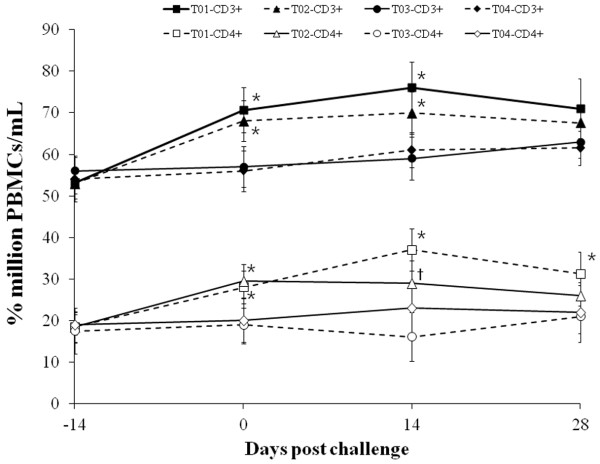
**Lymphocyte subsets analysis in the different groups; CD3**^**+**^**(■) and CD4**^**+**^**(□) from vaccinated challenged (T01) animals, CD3**^**+**^**(▲) and CD4**^**+**^**(△) from vaccinated non-challenged (T02) animals, CD3**^**+**^**(●) and CD4**^**+**^**(◯) from non-vaccinated challenged (T03) animals, and CD3**^**+**^**(♦) and CD4**^**+**^**(◇) from non-vaccinated non-challenged (T04) animals.** Variation is expressed as the standard deviation. Significant difference (T01 and T02 vs. T03 and T04) is indicated at *p* value <0.05^*^. Significant difference (T02 vs. T03) is indicated at *p* value <0.05^†^.

Vaccinated challenged (T01) and vaccinated non-challenged (T02) animals showed an increase in the relative proportions of CD3^+^ and CD4^+^ cells at 0 dpc compared with non-vaccinated challenged (T03) and non-vaccinated non-challenged (T04) animals (*p* < 0.05). Vaccinated challenged (T01) animals showed an increase in the relative proportions of CD3^+^ and CD4^+^ cells at 14 dpc, and in the relative proportions of CD4^+^ cells at 28 dpc compared with non-vaccinated challenged (T03) and non-vaccinated non-challenged (T04) animals (*p* < 0.05). At 14 dpc, the relative proportions of CD3^+^ cells were significantly higher in vaccinated non-challenged (T02) animals than non-vaccinated challenged (T03) and non-vaccinated non-challenged (T04) animals (*p* < 0.05, Figure 5). The number of CD4^+^ cells correlated with the number of PCV2-specific IFN-γ-SCs in the blood (T01: r^2^ = 0.624, *p* = 0.025, T02: r^2^ = 0.589, *p* = 0.047, and T03: r^2^ = 0.527, *p* = 0.041). The number of CD4^+^ cells also correlated with the DTH response (T01: r^2^ = 0.597, *p* = 0.023 and T03: r^2^ = 0.176, *p* = 0.365).

### Histopathology and immunohistochemistry

The histopathological lymphoid lesion scores were significantly lower in the vaccinated challenged (T01) animals than in the non-vaccinated challenged (T03) animals (*p* < 0.05, Table 1). The histopathological lymphoid lesion scores correlated with the number of genomic copies of PCV2 in the blood (T01: r^2^ = 0.870, *p* = 0.041 and T03: r^2^ = 0.892, *p* = 0.023). The mean number of PCV2-positive cells per unit area of lymph node was significantly lower in vaccinated challenged (T01) animals than in non-vaccinated challenged (T03) animals (*p* < 0.01, Table 1). The PCV2 antigen scores correlated with the number of genomic copies of PCV2 in the blood (T01: r^2^ = 0.855, *p* = 0.038 and T03: r^2^ = 0.872, *p* = 0.047).

## Discussion

Quantitation of the PCV2 viremia could predict PCV2 infection status. Several studies have already shown that PCV2 DNA levels in serum are higher in pig with PMWS than in healthy, subclinically infected pigs [20-22]. Hence, the reduction of PCV2 viremia by the PCV2 vaccine plays a critical role in controlling PCV2 infection. In the present study, the reformulated inactivated chimeric PCV1-2 vaccine is able to induce PCV2-specific NA and IFN-γ-SCs in vaccinated animals. This protective immunity induced by the reformulated inactivated chimeric PCV1-2 vaccine correlated with the reduction of PCV2 viremia in pigs challenged experimentally with solely PCV2 as induced by other commercial PCV2 vaccines did [10,23]. However, the presence of anti-PCV2 IgG antibodies did not correlate with the reduction of PCV2 viremia. The reformulated inactivated chimeric PCV1-2 vaccine also reduced the PCV2 load in nasal shedding in vaccinated animals, thereby decreasing the risk of transmission to other pigs via a nasal route and decreasing the amount of PCV2 circulating among the pigs.

IFN-γ, which is produced by PCV2-specific IFN-γ-SCs, is a key immunoregulatory cytokine that controls the differentiation of naïve CD4^+^ into CD4^+^ cells and mediates cellular immunity against viral infections [24]. Our results are further supported by the observation that elevated numbers of CD4^+^ cells are seen in vaccinated animals only. Lymphocyte subset analysis indicated that the numbers of CD3^+^ and CD4^+^ cells increased in vaccinated animals but the numbers of CD4^+^ cells decreased transiently in non-vaccinated animals. The selective loss of CD3^+^ and CD4^+^ cells that is observed in pigs with PMWS [25,26] may impair the immune system in the pigs and result in co-infections with other viral and bacterial pathogens; co-infections are frequently observed in pigs with PMWS under field conditions [27,28]. Because CD4^+^ cells promote a DTH response [29], the DTH response observed in only vaccinated animals also supports a CD4^+^ cell-associated protective cellular immune response that is induced by the reformulated inactivated chimeric PCV1-2 vaccine. PCV2-specific memory T lymphocytes induced by this chimeric PCV1-2 vaccine mount DTH reactions in response to intradermal injection of the PCV2 antigen. Hence, the induction of PCV2-specific NA and IFN-γ-SCs by the reformulated inactivated chimeric PCV1-2 vaccine is the important protective immune response that leads to reduce the PCV2 viremia and control the PCV2 infection.

Well-controlled experimental studies are necessary to elucidate the protective humoral and cellular immune response induced by the reformulated inactivated chimeric PCV1-2 vaccine to reduce PCV2 viremia because the results can be affected by factors such as environment, feed, pig source, immune status and inoculums. Under field conditions, PCV2 continues to circulate among pigs within the herd and the possibility of exposure and re-exposure to the virus by horizontal transmission occurs once one animal becomes infected. To our knowledge this is the first demonstration of protective humoral and cellular immunity induced by the reformulated inactivated chimeric PCV1-2 vaccine and its effect on reduction of PCV2 viremia by vaccination.

## Conclusions

The reformulated inactivated chimeric PCV1-2 vaccine is able to induce PCV2-specific NA and IFN-γ-SCs in vaccinated animals. This protective immunity induced by the reformulated inactivated chimeric PCV1-2 vaccine correlated with the reduction of PCV2 viremia in pigs challenged experimentally with solely PCV2.

## Competing interests

The authors state that there are no competing interests related to the present study.

## Authors’ contributions

HWS performance of the experimental trials, data analysis and writing of the manuscript, YO, KH and CP preparation of the inoculum and lab analysis, CC development of protocol, design of the study, review of the final manuscript, approval for publication. All authors read and approved the final manuscript.
